# Parthenolide ameliorates colon inflammation through regulating Treg/Th17 balance in a gut microbiota-dependent manner

**DOI:** 10.7150/thno.43716

**Published:** 2020-04-06

**Authors:** Yao-Jiang Liu, Bo Tang, Feng-Chao Wang, Li Tang, Yuan-Yuan Lei, Ya Luo, Sheng-Jie Huang, Min Yang, Ling-Yi Wu, Wei Wang, Shuang Liu, Shi-Ming Yang, Xiao-Yan Zhao

**Affiliations:** 1 Department of Gastroenterology, Xinqiao Hospital, Third Military Medical University, Chongqing 400037, China; 2 Institute of Combined Injury, State Key Laboratory of Trauma, Burns and Combined Injury, College of Preventive Medicine, Third Military Medical University, Chongqing 400037, China; 3 Department of Gastroenterology, The second Affiliated Hospital of Chongqing Medical University, Chongqing, 400010, China; 4 Department of Gastroenterology, Affiliated Hospital of North Sichuan Medical College, Nanchong, 637000, China; Yao-Jiang Liu and Bo Tang contributed equally to this work.

**Keywords:** Inflammatory bowel disease, Parthenolide, Gut microbiota, SCFAs, Treg/Th17 balance

## Abstract

Inflammatory bowel disease (IBD) is a global health problem in which gut microbiota dysbiosis plays an important pathogenic role. However, the current drugs for IBD treatment are far from optimal. Previous researches indicated that parthenolide (PTL) had not only anti-cancer properties but also strong anti-inflammatory activities.

**Rationale**: To investigate the protective effect of PTL on colon inflammation and demonstrate the underlying gut microbiota-dependent mechanism.

**Methods:** Colon inflammation severity in mouse model was measured by body weight change, mortality, colon length, disease activity index (DAI) score, H&E staining and colonoscopy evaluation. Gut microbiota alteration and short-chain fatty acids (SCFAs) production were analyzed through 16S rRNA sequencing and targeted metabolomics. Luminex cytokine microarray and Enzyme-linked immunosorbent assay (ELISA) were conducted to measure the colon cytokines profile. The frequency of immune cells in lamina propria (LP) and spleen were phenotyped by flow cytometry.

**Results**: The PTL-treated mice showed significantly relieved colon inflammation, as evidenced by a reduction in body weight loss, survival rate, shortening of colon length, DAI score, histology score and colonoscopy score. Notably, when the gut microbiota was depleted using antibiotic cocktails, the protective effect of PTL on colon inflammation disappeared. PTL treatment downregulated the level of proinflammatory cytokines, including IL-1β, TNF-α, IL-6, and IL-17A and upregulated the immunosuppressive cytokine IL-10 in colon tissue. 16S rRNA sequencing indicated that PTL-treated mice exhibited much more abundant gut microbial diversity and flora composition. Targeted metabolomics analysis manifested the increased SCFAs production in PTL-treated mice. Additionally, PTL administration selectively upregulated the frequency of colonic regulatory T (Treg) cells as well as downregulated the ratio of colonic T helper type 17 (Th17) cells, improving the Treg/Th17 balance to maintain intestinal homeostasis. Gut microbiota depletion and fecal microbiota transplantation (FMT) was performed to confirm this gut microbiota-dependent mechanism.

**Conclusions**: PTL ameliorated colon inflammation in a gut microbiota-dependent manner. The underlying protective mechanism was associated with the improved Treg/Th17 balance in intestinal mucosa mediated through the increased microbiota-derived SCFAs production. Collectively, our results demonstrated the role of PTL as a potential gut microbiota modulator to prevent and treat IBD.

## Introduction

Inflammatory bowel disease (IBD) mainly includes two phenotypes, that is, Crohn's disease (CD) and ulcerative colitis (UC), which is a chronic recurrent inflammatory disease of the gastrointestinal tract. Associated with substantial morbidity, decreased quality of life, and colitis-correlated colorectal cancer (CAC) progression, IBD has increasingly emerged as a public health challenge worldwide 1, 2. However, current drugs for IBD treatment are far from optimal, and patients have to face lifelong therapy and debilitation. Therefore, alternative safe and effective new drugs need to be developed.

The pathogenesis of IBD consists of a combination of genetic susceptibility, environmental exposure, and dysregulated immune responses to gut microbiota alterations 3-5. Emerging evidence has indicated that perturbations in the gut microbiome are an essential factor triggering inflammation in IBD rather than merely a consequence of chronic inflammation 6, 7. Specifically, gut microbiota dysbiosis in patients with IBD is usually characterized by the loss of beneficial commensal microflora, the expansion of pathogenic bacteria, and the reduced overall biodiversity of the microbial ecosystem 8, 9. It is widely accepted that gut microbiota disorders could affect the regulatory T (Treg)/T helper type 17 (Th17) balance in IBD, triggering exaggerated inflammatory responses in the intestine^10^, ^11^. Th17 cells, characterized by the production of proinflammatory cytokines (i.e., IL-17A), are a major contributor in multiple autoimmune diseases, including IBD 12, 13. In contrast, Treg cells have been identified as dedicated suppressors of diverse immune responses and inflammations through the secretion of the anti-inflammatory cytokine IL-10 14, 15. These two cells play opposite roles in the inflammatory responses to jointly maintain intestinal immune homeostasis. However, during gut microbiota dysbiosis, this balance is disturbed due to the excessive growth of pathogenic bacteria and reduced gut microbiota diversity, causing the Treg/Th17 balance to tend toward Th17 cells 16, 17. Short-chain fatty acids (SCFAs), mainly including acetate, propionate, and butyrate, are metabolic end-products produced in the gastrointestinal tract by fermentation of nondegradable carbohydrates and proteins, which have been shown to promote Treg cell differentiation and inhibit intestinal inflammation 18, 19. Therefore, gut microbiota diversity and microbiota-derived SCFAs altering the Treg/Th17 balance play important roles in the inflammatory responses of IBD 20, 21. The above evidence proves that the modulation of gut microbiota may be a promising IBD treatment strategy. Studies have shown that probiotics (such as *Bifidobacterium fragilis*) could increase Treg cells to induce IL-10 production, thereby improving the mucosal immune responses of IBD 22-24. Multiple studies have proven that natural plant extracts could act as effective and safe gut microbiota regulators. For instance, polysaccharides extracted from Hirsutella sinensis mycelium (HSM) can reverse obesity-induced gut microbiota dysbiosis and reduce insulin resistance and inflammatory responses 25. The natural plant camu camu (*Myrciaria dubia*) prevents obesity by altering the gut microbiota and increasing energy expenditure in diet-induced obese mice 26.

Parthenolide (PTL), a sesquiterpene lactone originally extracted from the shoots of the plant Feverfew (*Tanacetum balsamita*), has shown potent anti-cancer, anti-inflammatory and even anti-bacterial biological activities 27-29. As a natural compound, the anti-cancer activity of parthenolide has been suggested to be due to its pro-apoptotic actions, which would occur through the activation of p53 and the increased production of reactive oxygen species (ROS) exclusively in tumor cells, while at the same time having no effect on normal cells, making PTL a promising anti-cancer drug 30. However, although some studies have reported the anti-inflammatory effects and mechanism of PTL *in vitro* 31, the anti-inflammatory and anti-bacterial effects *in vivo*, especially in IBD, have rarely been described. Given gut microbiota dysbiosis plays an important role in the pathogenesis of IBD, we sought to analyze the potential impact of the administration of PTL on gut microecosystem as well as IBD in dextran sodium sulfate (DSS)-induced colitis mice.

In this study, we investigated for the first time the effect of PTL administration on colon inflammation. To identify the role of gut microbiota in mediating the protective effect of PTL on DSS-induced colitis, gut microbiota depletion and fecal microbiota transplant (FMT) were conducted. Finally, the underlying gut microbiota-dependent mechanism was also explored.

## Materials and Methods

### Animals

C57BL/6 (wild type; WT) mice (male; 8 to 10 weeks of age; weighing 18 to 22 g) were purchased from the Animal Center of Xinqiao Hospital. Throughout the acclimatization and study periods, all animals were maintained on a 12 h light-dark cycle (21 ± 2°C with a relatively constant humidity of 45 ± 10%) under specific pathogen-free conditions and had access to food and water ad libitum. All animal experimental protocols were performed following the guidelines of the National Institutes of Health guide for the care and use of Laboratory Animals, approved by the Third Military Medical University Institutional Animal Care and Use Committee.

### DSS-induced colitis

To induce acute experimental colitis, mice were administered 1.5-3.0% (w/v) dextran sodium sulfate (DSS, molecular weight, 36-50 kDa; MP Biomedicals, UK) in their drinking water ad libitum for 7 days followed by 7 days normal water. Parthenolide (PTL) (10 mg/kg) suspended in saline was administered by intraperitoneal (i.p.) injection in PTL+DSS group mice from day 0 to day 14 until the end of experiments. Mice in DSS group underwent i.p. injection of normal saline as a negative control. To avoid abdominal infection caused by i.p. injection, the procedures were strictly performed under sterile conditions. In all colitis models, mice were checked daily for morbidity and body weight was recorded. Each mouse was scored daily for pathological features, including stool consistency, presence of blood stool, and body weight loss. Individual scores were combined to generate the Disease Activity Index (DAI) which was calculated daily for each mouse. The maximum score was 12 based on assigning a 0-4 scoring system for following parameters (Supplementary Table S1).

### Histopathology

The colons were emptied of fecal contents and opened longitudinally along the mesenteric border and formed a Swiss-Roll from the proximal to the distal end, then placed in 10% neutral buffered formalin for 24 h. The Swiss-rolls were transferred to 70% ethanol and then processed to paraffin-embedded blocks to generate 5-µm-thick sections for hematoxylin and eosin (H&E) staining. Slices were evaluated by the experienced pathologist in a blinded manner and histological scores were assessed based on the following parameters according to previous research: inflammation, epithelial defects, crypt atrophy, dysplasia/neoplasia, and the area affected by dysplasia 32, 33.

### Colonoscopy

Colonoscopy was performed on experimental mice using a high‐resolution mouse video endoscopic system (KARL STORZ, Tuttlingen, Germany). Anesthetize mice using 1.5% to 2.0% isoflurane anesthesia, then inflate the colon with air to visualize 3 cm of the proximal colon. The severity of colitis was scored in a blinded manner based on the following parameters: colon translucency (0-3), presence of fibrin attached to the bowel wall (0-3), granular aspect of the mucosa (0-3), morphology of the vascular pattern (0-3), stool characteristic: normal to diarrhea (0-3), presence of blood in the lumen (0-3), generating a maximum score of 18.

### Fecal genomic DNA extraction and 16S-rRNA sequencing

Fecal genomic DNA was extracted from 0.1 g frozen fecal samples using an E.Z.N.A.® soil DNA Kit (Omega Bio-Tek, Norcross, GA, U.S.) according to the manufacturer's protocol. The DNA concentration and purification were measured using a NanoDrop 2000 spectrophotometer (Thermo Scientific, Wilmington, USA), and the DNA quality was detected by 1% agarose gel electrophoresis. Amplicon libraries covering the V3-V4 hypervariable regions of the bacterial 16S-rDNA gene were amplified using primers 341F: 5′-ACTCCTACGGGRSGCAGCAG-3′, and 806R: 5′-GGACTACVV GGGTATCTAATC-3′. PCR was performed in a 20 μl mixture containing 4 μl of 5 × FastPfu Buffer, 2 μl of 2.5 mM dNTPs, 0.8 μl of each primer (5 μM), 0.4 μl of FastPfu Polymerase and 10 ng of template DNA. PCR was conducted with an initial denaturation for 3 min at 95 °C, followed by 27 cycles of 30 s at 95 °C, 30 s for annealing at 55 °C, and 45 s for elongation at 72 °C, and a final extension at 72 °C for 10 min. The reactions were performed on a thermocycler PCR system (GeneAmp 9700, ABI, USA). All PCR products were purified using an AxyPrep DNA Gel Extraction Kit (Axygen Biosciences, Union City, CA, USA) and quantified using QuantiFluor™-ST (Promega, USA). Purified and pooled amplicon libraries were paired-end sequenced (2 × 300) on the Illumina MiSeq platform (Illumina, San Diego, USA) according to the standard protocols by Majorbio Bio-Pharm Technology Co., Ltd. (Shanghai, China).

### Cytokines microarray assay

The colon explant cultures were performed according to the previous description 34. The longitudinally opened colon was washed in ice-cold PBS containing 2 × penicillin-streptomycin. Since different areas within the colon showed differences in the severity of colitis, we used a 3 mm skin punch tool to generate three to four defined circular biopsy samples from the same distal colon area for specific analysis. A single biopsy sample was transferred to a well of a 48-well plate containing 0.5 mL of sterile cell culture medium and incubated for 15 h in a cell incubator at 37 °C. The supernatant was collected, centrifuged to remove debris, and the cytokines were evaluated by the Luminex Bio-Plex system according to the manufacturer's instructions.

### Enzyme-linked immunosorbent assay (ELISA)

Colon tissue was weighed and put into 900 mL normal saline followed by ultrasonic trituration and centrifugation at 3000 rpm for 10 min to obtain colon tissue homogenate. Cytokines including IL-10 (Dakewe, Cat. No.1211002), IL-17A (Dakewe, Cat. No. 1211702), IL-1β (Dakewe, Cat. No.1210122), IL-6 (R&D, Cat. No. M6000B), TNF-α (R&D, Cat. No. MTA00B) in the colon tissue homogenate were measured by ELISA Kits according to the manufacturer's instructions. Capture antibodies (1:200) were coated in the plate overnight at 4 ℃. After blocking, samples were incubated for 2 h at room temperature, followed by incubation with detection antibodies (1:200) for 1 h. Subsequently, streptavidin conjugated with horseradish peroxidase was added, and the substrate was added after 30 min. Finally, the absorbance value was detected using a microplate reader. The concentrations of cytokines were obtained according to the standard curves.

### Targeted SCFAs quantitative analysis

Fecal samples (10 mg) were supplemented with 10 μl of internal standards (0.0125 μl/μl 2-methyl butyric acid, Sigma-Aldrich) and 500 μl of methanol, and then extracted according to the manufacturer's protocol (Majorbio Bio-Pharm Technology Co., Ltd., Shanghai, China). The extracted samples were detected using a 6890A-5973C GC-MS system (Agilent Technologies, Santa Clara, CA). SCFAs standards were mixtures of acetate, propionate, butyrate, isobutyrate, valerate, and isovalerate. All the standards, excluding isovalerate (Sigma-Aldrich), were purchased from Merck (Darmstadt, Germany).

### Fecal microbiota transplantation (FMT)

FMT was performed according to the modified method described previously 35, 36. Briefly, 8- to 10-week-old male C57BL/6 mice received antibiotic cocktails (vancomycin, 100 mg/kg; neomycin sulfate 200 mg/kg; metronidazole 200 mg/kg; and ampicillin 200 mg/kg) intragastrically once a day for 5 days for gut microbiota depletion. Feces from donor mice (PTL+DSS and DSS groups) were collected and resuspended in PBS at 0.125 g/mL, then 0.15 mL of this suspension was administered to mice by oral gavage once a day for continuous 5 days.

### Flow cytometric analysis

For the staining of intracellular cytokines IL-10 and IL-17A, mononuclear cell isolation was first stimulated with ionomycin (Abcam, Cat. No. ab120370, 750 ng/mL) and PMA (Abcam, Cat. No. ab120297, 50 ng/mL) for 2 h in 5% CO_2_ at 37°C. After GolgiStop protein transport inhibitor (BD, Cat. No. 554724, 0.7μl/mL) was added into cell suspension, cells were incubated for another 3 h in 5% CO_2_ at 37 °C. Subsequently, cells were stained with live/dead dye and surface markers for 30 min at 4 °C in the dark, and then fixed and permeabilized the cells with Fixation/ Permeabilization working solution and Permeabilization Buffer for 20 min at RT in the dark. Finally, stained cells with the anti-IL-10 or anti-IL-17A antibody. To analyze the percentage of transcription factor Foxp3^+^ expression, mononuclear cells isolated were first stained with live/dead dye (Invitrogen, Cat. No. L34975) and surface markers of CD45, CD4, CD25 for 30 min at 4 °C in the dark. Subsequently, cells were fixed and permeabilized with Fixation/Permeabilization working solution and Permeabilization Buffer for 20 min at RT in the dark. Finally, stained cells with anti-Foxp3 antibody.

### FACS antibodies

Antibodies for FACS included PerCP-labeled anti-mouse CD45 antibody (Biolegend, Cat. No. 103130), Brilliant Violet 510-labeled anti-mouse CD4 antibody (Biolegend, Cat. No. 100559), PE-labeled anti-mouse CD25 antibody (Biolegend, Cat. No. 102007), FITC-labeled anti-mouse IL-10 antibody (Biolegend, Cat. No. 505006), PE-labeled anti-mouse-IL-17A antibody (Biolegend, Cat. No. 506904), Alexa Fluor 647-labeled anti-mouse Foxp3 antibody and isotype-matched control (Biolegend, Cat. No. 320014), BV421-labeled anti-mouse Foxp3 antibody and isotype-matched control (BD, Cat. No. 562996).

### Statistical analysis

Based on whether the data were normally distributed and the number of tested groups for comparison, the levels of significance were determined by appropriate statistical analysis. Student's t-test (unpaired, two-tailed) was used to determine levels of significance for comparisons between two groups by using Graphpad Prism 7.0 software (GraphPad Software Inc., San Diego, USA). Results are shown as mean ± SD; statistical significance is indicated as follows: *p < 0.05; ** p < 0.01; *** p < 0.001 and NS means no significance. Bacterial alpha-diversity was determined by sampling-based OTUs analysis and presented by Observed OTU, Chao index, Shannon index, and Simpson index, which was calculated using the R program package 'vegan'. Principal coordinates analysis (PCoA) was conducted by R package (http://www.R-project.org/) to display microbiome beta-diversity between samples. The Bray-Curtis metric distances, Unweighted-Unifrac distance, and Weighted-Unifrac distances were calculated with the phyloseq package. Bacterial taxonomic analyses and comparison including bacterial phylum, class, order, family, genus levels were conducted between two groups using the Wilcoxon rank-sum test. The predominance of bacterial communities between groups were analyzed by linear discriminant analysis (LDA) effect size (LEfSe) method (http://huttenhower.sph.harvard.edu/lefse/) e/). Based on the normalized relative abundance matrix, features with significantly different abundances between assigned taxa were determined by LEfSe with the Kruskal-Wallis rank-sum test (*p* < 0.05) and LDA was used to assess the effect size of each feature (LDA score (log10) = 3.6 as cut-off value).

## Results

### PTL administration ameliorated DSS-induced colitis

**Figure 1:**
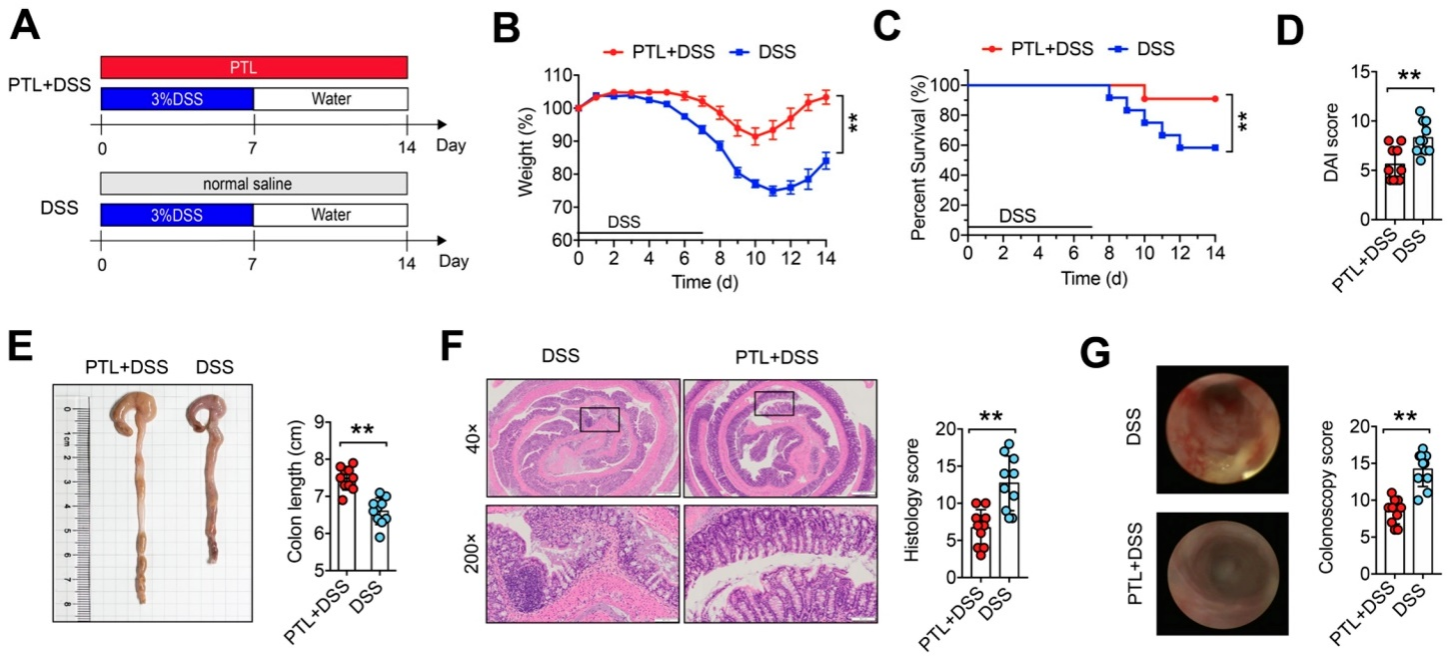
PTL treatment ameliorated DSS-induced experimental colitis. (A) To assess experimental colitis and repair, WT mice aged between 8 and 10 weeks from two groups were given oral administration of 3.0% DSS for 7 days followed by normal water drinking for a further 7 days. The DSS and PTL+DSS groups were intraperitoneal injection with 100 μl saline or 100 μl PTL solution from Day 0 to Day 14, respectively. (B) Body weight change. (C) Survival. (D) Disease activity index (DAI) score. (E) Representative pictures of colon gross appearance and colon length. (F) Representative microscopic pictures of H&E staining (40x and 200x magnification) and histology score. (G) Representative colonoscopy images and colonoscopy score. (A-G) n = 10 mice per group, mean values ± SD are presented, *p* values were calculated using Unpaired T-test, * *p* < 0.05, ** *p* < 0.01, *** *p* < 0.001. Data are pooled from three independent experiments with n = 10 mice per group.

To investigate whether PTL has a therapeutic effect on IBD, DSS-induced colitis in a mouse model was employed. Mice were treated with 3.0% DSS in their drinking water ad libitum for 7 days followed by 7 days of normal water, while PTL suspended in saline was administered by intraperitoneal injection in PTL+DSS group mice from day 0 to day 14 (Figure 1A). Compared with the DSS group, PTL administration significantly ameliorated DSS-induced colitis, as evidenced by the markedly reduced weight loss (Figure 1B), decreased mortality (Figure 1C) and significant relief of colonic shortening (Figure 1E). Furthermore, DAI score based on the assessment of stool consistency, bloody stool, and weight loss displayed a consistent tendency with the above results (Figure 1D). H&E staining and colonoscopy were performed to systematically evaluate colonic mucosa injury. Compared with DSS-treated group mice showing loss of crypts, infiltration of mononuclear cells, severe mucosal damage, and higher histology score, PTL+DSS group mice exhibited less inflammatory cell infiltration, relatively intact colonic architecture, less mucosal damage and lower histology score (Figure 1E). Additionally, DSS group mice showed higher colonoscopy score (Figure 1G), specifically in granular intestinal lumen surfaces, the presence of blood, ulceration formation and plenty of inflammatory fibrin exudation, which can be manifested vividly in colonoscopy video (Supplementary Video S1). Conversely, PTL+DSS group mice displayed relatively smooth lumen surfaces, clear vascular morphology and the absence of blood and representative colonoscopy video was shown in Supplementary Video S2. Together, these results indicated that PTL treatment significantly ameliorated DSS-induced colitis.

### PTL alleviated colitis in a gut microbiota-dependent manner

**Figure 2:**
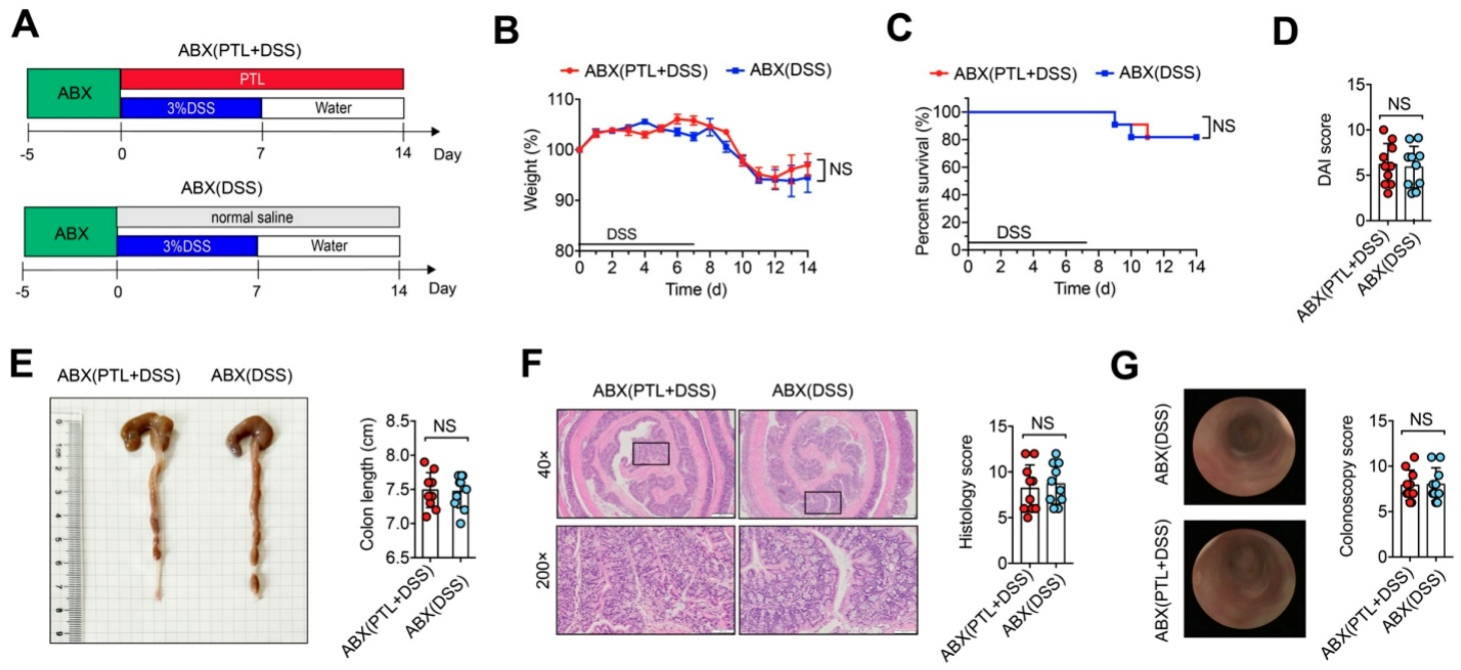
The protective effect of PTL against DSS-induced colitis disappeared after gut-microbiota depletion. (A) WT mice were put on a course of intragastric antibiotics administration for 5 days for gut microbiota depletion prior to DSS treatment. The ABX(DSS) and ABX(PTL+DSS) groups were intraperitoneal injection with 100 μl saline or 100 μl PTL solution from Day 0 to Day 14, respectively. (B) Body weight change. (C) Survival. (D) DAI score. (E) Representative pictures of colon gross appearance and colon length. (F) Representative microscopic pictures of H&E staining (40x and 200x magnification) and histology score. (G) Representative colonoscopy images and colonoscopy score. (A-G) n = 10 mice per group, mean values ± SD are presented, *p* values were calculated using Unpaired T-test, * *p* < 0.05, ** *p* < 0.01, *** *p* < 0.001. Data are pooled from three independent experiments with n = 10 mice per group.

Although the precise etiology and pathogenesis of IBD remain unclear, abundant evidence has indicated that dysregulated responses to intestinal microbiota alteration contribute to the occurrence of IBD, making the gut microbiota a new target for IBD treatment. To investigate whether gut microbiota participated in the protective effect of PTL against DSS-induced colitis, wild-type mice were gavaged using the quadruple antibiotic cocktails for gut microbiota depletion before DSS treatment. Since there is reported enhanced sensitivity of gut microbiota-depleted mice to DSS 37, we used a low dose of 1.5% DSS in drinking water for 7 days followed by normal water drinking for an additional 7 days for antibiotic-treated mice (Figure 2A). Strikingly, ABX(DSS) group mice and ABX(PTL+DSS) group mice displayed indistinguishable weight loss (Figure 2B), mortality (Figure 2C), colon length (Figure 2E), DAI score (Figure 2D), histology score (Figure 2F) and colonoscopy score (Figure 2G) following gut microbiota depletion. Representative microscopic H&E staining pictures and colonoscopy images are shown in Figure 2F-G. These results demonstrated that the anti-inflammatory protective effect of PTL on colon inflammation was gut microbiota-dependent.

### Fecal microbial transplantation mitigated colitis

**Figure 3:**
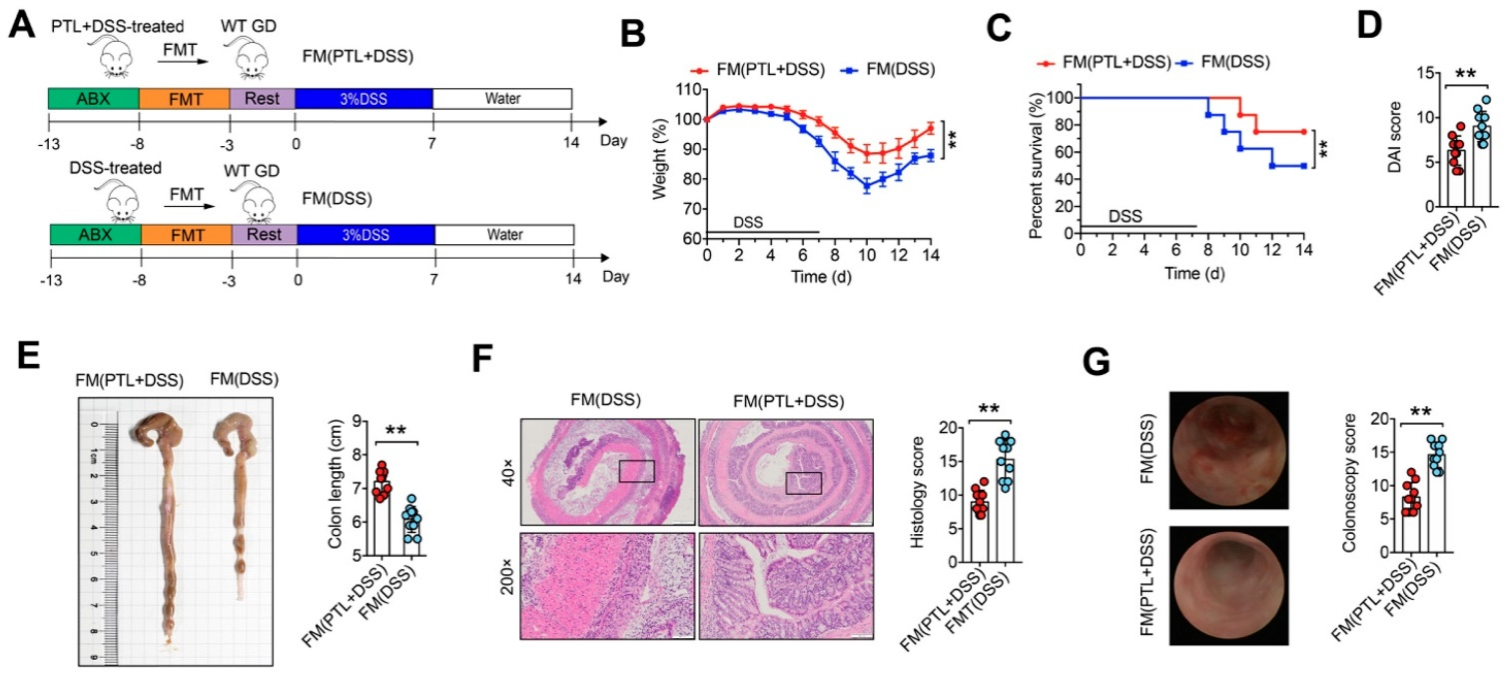
Fecal microbial transplantation mitigated DSS-induced experimental colitis. (A) WT mice were put on a course of intragastric antibiotics administration for 5 days for gut microbiota depletion prior to FMT. After 5 days of intragastric FMT and 3 days of intestinal rest, mice received 7 days treatment with 3.0% DSS following 7 days normal drinking water. (B) Body weight change. (C) Survival. (D) DAI score. (E)Representative pictures of colon gross appearance and colon length. (F) Representative microscopic pictures of H&E staining (40x and 200x magnification) and histology score. (G) Representative colonoscopy images and colonoscopy score. (A-G) n = 10 mice per group, mean values ± SD are presented, *p* values were calculated using Unpaired T-test, * *p* < 0.05, ** *p* < 0.01, *** *p* < 0.001. Data are pooled from three independent experiments with n = 10 mice per group.

To further confirm whether the alleviation of colitis in PTL-treated mice depends on gut microbiota, we performed fecal microbiota (FM) transplantation experiments in which gut microbiota-depleted wild-type (GD WT) mice were reconstituted with the microbiota of DSS-treated mice (FM(DSS) → GD WT) and PTL-treated mice (FM(PTL+DSS) → GD WT) via intragastric administration once a day for 5 days (Figure 3A). For better flora colonization, all experimental mice were gavaged with the antibiotic cocktails to create gut microbiota depletion conditions before FM. Transfer of FM from a PTL-treated donor into a GD WT host mouse (FM(PTL+DSS) → GD WT) resulted in significantly less colon inflammation, as evidenced by the decreased weight loss (Figure 3B), mortality (Figure 3C), and DAI score (Figure 3D) but greater colon length (Figure 3E) than did the transfer of FM from a DSS-treated donor (FM(DSS) → GD WT). H&E staining of colon tissue from FM(PTL+DSS) → GD WT showed less inflammatory cell infiltration, relatively intact colonic architecture, less mucosal damage and lower histology score compared with colons from FM(DSS) → GD WT (Figure 3F). The colonoscopy images and colonoscopy score results were consistent with the above observations (Figure 3G). These FMT results indicated that the intestinal microbiota in PTL-treated mice was responsible for alleviated colon inflammation.

### PTL treatment significantly altered gut microbiota diversity and composition

**Figure 4:**
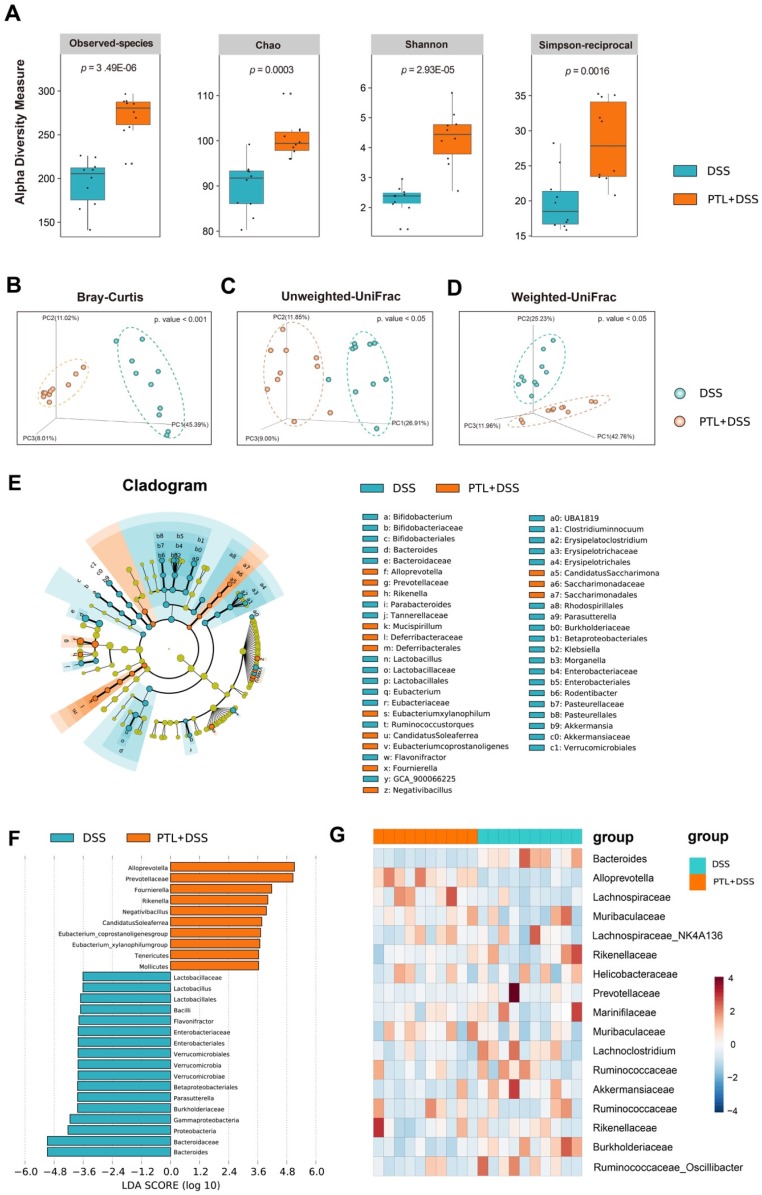
PTL treatment significantly altered the gut microbiota diversity and composition. (A) Alpha diversity boxplot (observed species, Chao, Shannon and Simpson reciprocal). (B) Principal coordinate analysis (PCoA) using Bray-Curtis metric distances of beta diversity. (C) PCoA using Unweighted-UniFrad of beta diversity. (D) PCoA using Weighted-UniFrad of beta diversity. (E) Taxonomic cladogram from LEfSe, depicting taxonomic association from between microbiome communities from DSS and PTL+DSS groups. Each node represents a specific taxonomic type. Yellow nodes denote the taxonomic features that are not significantly differentiated between DSS and PTL+DSS groups. Orange nodes denote the taxonomic types with more abundance in PTL+DSS than in DSS group, while the blue nodes represent the taxonomic types more abundant in DSS group. (F) LDA score computed from features differentially abundant between DSS and PTL+DSS groups. The criteria for feature selection is log LDA score > 3.6. (G) Heatmap of selected most differentially abundant features at the genus level. The blue color represents less abundant, white color represents intermediate abundance and red represents the most abundant. (A-G) n = 10 samples per group. Each symbol represents an individual mouse. Data are pooled in one independent experiment with n = 10 mice per group.

To determine whether PTL treatment altered the microbiome, we performed high-throughput gene-sequencing analysis of 16S rRNA in fecal bacterial DNA isolated from PTL+DSS group and DSS group mice. We originally measured gut microbial alpha diversity using a generalized linear model through different methodologies. Consistently, different indices including observed taxonomic units (OTUs), Chao, Shannon and Simpson manifested similar tendencies and found that PTL-treated mice harbored a microbiota with significantly higher alpha diversity relative to that of the DSS-treated group (*p* < 0.005, *p* <0.005, *p* < 0.005, and *p* < 0.005 for each alpha diversity index) (Figure 4A). To extend our understanding of the role of microbiome diversity, we performed beta-diversity to generate a principal coordinate analysis (PCoA) using Bray-Curtis metric distance, Unweighted-UniFrac distance, and Weighted-UniFrac distance algorithms. An apparent clustering separation between OTUs revealed the different community structures between the two groups, suggesting that these communities are distinct in terms of their compositional structure (Figure 4B-D).

Subsequently, we assessed the landscape of the gut microbiota in all available samples to further investigate the potential composition difference between the PTL+DSS group and the DSS group. In terms of bacterial composition at the phylum level, all samples shared similar taxonomic communities and exhibited a relatively high abundance of the phyla *Bacteroidetes* and *Firmicutes* (Supplementary Figure S1A). *Bacteroidetes* were the most predominant phylum, accounting for 64.28% and 61.21% of the gut microbiota in the PTL+DSS and DSS groups, respectively (*p* = 0.80) (Supplementary Figure S1B). *Firmicutes* were the second most predominant phylum in both groups, with proportions of 29.45% and 28.40%, respectively (*p* = 0.85) (Supplementary Figure S1C). Consistently, the ratio of *Firmicutes* to *Bacteroidetes* (F/B) displayed no statistically significant difference between the two groups (*p* > 0.05) (Supplementary Figure S1D). Taxonomic compositions between the PTL+DSS group and DSS group were also compared at the class/order/family level (Supplementary Figure S2A-S2C). At the genus level, the two groups displayed differential biological compositions (Supplementary Figure S3A). Bacterial genera that were present at a relative abundance of > 1% were analyzed. In particular, the genus *Alloprevotella* (30.75% vs 4.08%, respectively; *p* < 0.001, q < 0.001) displayed a relatively high abundance in the PTL+DSS group, whereas the genus *Bacteroides* (4.40% vs 29.58%, respectively; *p* < 0.001, q < 0.001) were less abundant in the PTL+DSS group than in the DSS group (Supplementary Figure S3B). The relative abundance of *Alloprevotella* and* Bacteroides* in each sample between two groups were displayed in Supplementary Figure S3C-D.

To confirm which bacterium was altered by PTL treatment and in turn affected the disease progression against DSS-induced colitis, we performed high-dimensional class comparisons using the linear discriminant analysis (LDA) of effect size (LEfSe) that detected marked differences in the predominance of bacterial communities between the two groups (Figure 4E-F). According to the analysis results, *Bacteroidaceae* (the family and the genus *Bacteroides*) and the genus *Lactobacillus* (from the class *Bacilli* to the family *Lactobacillaceae*) were the key types of bacteria contributing to gut microbiota dysbiosis in the DSS group.

Nevertheless, *Prevotellaceae* (the family and the genus *Alloprevotella*), *Rikenella*, and *Fournierella* displayed a relative enrichment in the PTL+DSS group, which might be associated with the PTL-mediated alleviation of colitis. Within these, the genus *Alloprevotella* had the highest LDA score of 5.33 (*p* = 0.0002), followed by* Fournierella* with an LDA score of 4.93 (*p* = 0.0009). Based on the OTU abundance at the genus level, we also organized a comparison heatmap for the analysis of gut microbiota between the two groups (Figure 4G). Similarly, the genus *Alloprevotella* displayed a relatively high abundance in the PTL+DSS group, while the genus *Bacteroides* was significantly enriched in the DSS group, which was consistent with the LEfSe analysis results. Collectively, PTL treatment significantly altered the gut microbiota diversity and composition.

### PTL treatment increased metabolite SCFAs

**Figure 5:**
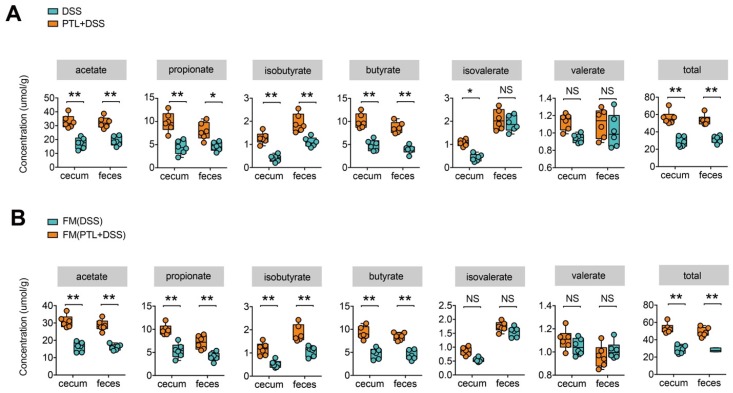
PTL treatment increased the production of microbial metabolites SCFA. (A) SCFA concentration from cecal content and feces between PTL+DSS and DSS groups. Group differences were tested with Wilcoxon tests, notable differences were as follows: acetate: cecum *p* < 0.01 & feces *p* < 0.01, propionate: cecum *p* < 0.01 & feces p = 0.03, isobutyrate: cecum *p* < 0.01 & feces *p* < 0.01, butyrate: cecum *p* < 0.01& feces *p* < 0.01, isovalerate: cecum *p* = 0.04 and feces *p* > 0.05, valerate : cecum *p* > 0.05 and feces *p* > 0.05, total SCFA: cecum p < 0.01 & feces p < 0.01. (B) SCFA concentration from cecal content and feces between FM(PTL+DSS) and FM(DSS) group. Acetate: cecum *p* < 0.01 & feces *p* < 0.01, propionate: cecum *p* < 0.01 & feces p < 0.01, isobutyrate: cecum *p* < 0.01 & feces *p* < 0.01, butyrate: cecum *p* < 0.01& feces *p* < 0.01, isovalerate: cecum *p* > 0.05 and feces *p* > 0.05, valerate : cecum *p* > 0.05 and feces *p* > 0.05, total SCFA: cecum p < 0.01 & feces p < 0.01. (A-B) n = 6 mice per group, mean values ± SD are presented, *p* values were calculated using Unpaired T-test, * *p* < 0.05, ** *p* < 0.01, *** *p* < 0.001. Each symbol represents an individual mouse. Data are pooled from three independent experiments with n = 6 mice per group.

Previous 16S rRNA sequencing analysis showed that gut microbiota in the PTL+DSS group displayed a predominance of the genera *Alloprevotella*, *Rikenella*, and *Fournierella*, which were associated with SCFAs metabolism 38-41. It is well known that gut microbiota metabolite SCFAs play a key role in the maintenance of intestinal homeostasis, and decreased SCFAs concentrations were observed in IBD patients as well as in DSS-induced colitis in mice 20, 42. To investigate whether the changes in bacterial communities had an impact on microbial metabolic output, the SCFAs concentrations in cecal contents and fecal samples were evaluated by a targeted metabolomics assay. Consistent with the changes in microbial community structure and compositions, the PTL+DSS and DSS groups had totally different SCFAs profiles (Figure 5A). The PTL+DSS group manifested higher amounts of acetate (cecum: *p* < 0.01 & feces: *p* < 0.01), propionate (cecum: *p* < 0.01 & feces: *p* = 0.03), isobutyrate (cecum: *p* < 0.01 & feces: *p* < 0.01), and butyrate (cecum: *p* < 0.01 & feces: *p* < 0.01) in both the cecum and feces. Although there were no significant differences in terms of isovalerate (cecum: *p* = 0.04 & feces: *p* > 0.05) and valerate levels (cecum: *p* > 0.05 & feces: *p* > 0.05), total SCFAs in the PTL+DSS group were significantly higher than those in the DSS group in both the cecum and feces (cecum: *p* < 0.01 & feces: *p* < 0.01).

To investigate whether the increased metabolite SCFAs production originated from gut microbiota alterations following PTL administration, targeted metabolite SCFAs productions were also conducted between FMT groups. As expected, the SCFAs production profile between FM(PTL+DSS) and FM(DSS) groups manifested relatively similar tendency to that of the PTL+DSS and DSS groups (Figure 5B). The concentrations of acetate (cecum: *p* < 0.01 & feces: *p* < 0.01), propionate (cecum: *p* < 0.01 & feces: *p* < 0.01), isobutyrate (cecum: *p* < 0.01 & feces: *p* < 0.01), and butyrate (cecum: *p* < 0.01 & feces: *p* < 0.01) in the FM(PTL+DSS) group were significantly higher than those in the FM(DSS) group in both the cecal and fecal samples. Total SCFAs in the FM(PTL+DSS) group were significantly higher than those in the FM(DSS) group in both the cecum and feces (cecum: *p* < 0.01 & feces: *p* < 0.01). Collectively, PTL treatment modulated gut bacterial community structures and compositions, which were associated with increased metabolite SCFAs levels.

### PTL administration downregulated pro-inflammatory cytokines and upregulated anti-inflammatory cytokines in DSS-induced colitis

**Figure 6:**
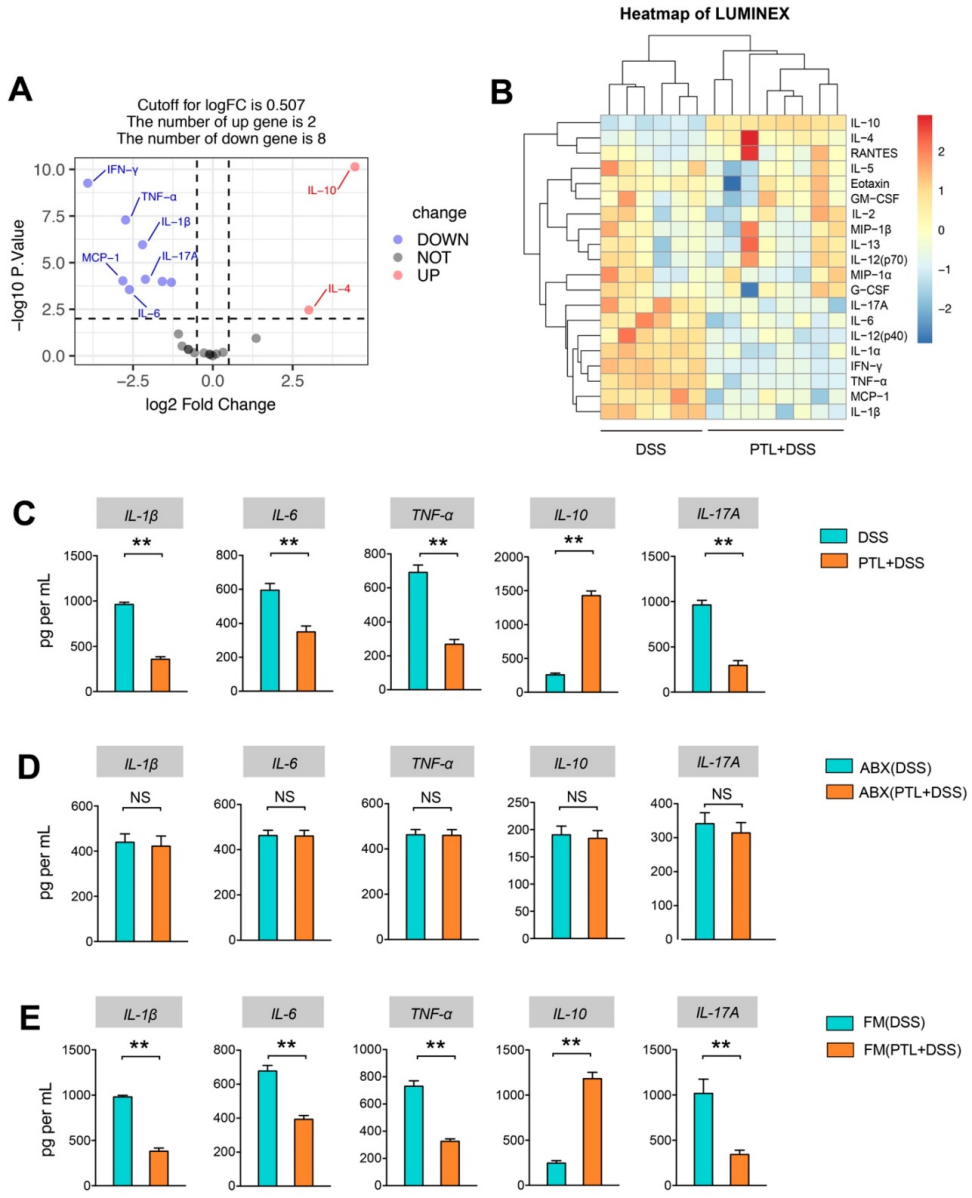
PTL treatment altered the cytokine profile in colitis mice. (A) Volcano plot of cytokine alteration from colon tissue supernatant as measured by Luminex bead-based immunoassays. (B) Heatmap representation of cytokine levels from colon tissue supernatant as measured by Luminex (PTL+DSS group, n = 8; DSS group, n = 6). Significance analysis of microarrays (SAM) was used to identify tissue supernatant proteins at significantly different levels between groups (q < 0.001, fold-change > 2). A hierarchically clustered (unsupervised, Euclidean distance) heatmap of the SAM-positive proteins is shown. The raw mean fluorescence intensity (MFI) of all proteins were log2 transformed prior to clustering. (C) IL-1β, IL-6, TNF-α, IL-10 and IL-17A cytokines levels in colon tissue homogenate were measured by ELISA between PTL+DSS and DSS groups. (D) IL-1β, IL-6, TNF-α, IL-10 and IL-17A cytokines levels in colon tissue homogenate were measured by ELISA between ABX(PTL+DSS) and ABX(DSS) groups. (E) IL-1β, IL-6, TNF-α, IL-10 and IL-17A cytokines levels in colon tissue homogenate were measured by ELISA between FM(PTL+DSS) and FM(DSS) groups. (C-E) n = 5 mice per group, mean values ± SD are presented, *p* values were calculated using Unpaired T-test, * *p* < 0.05, ** *p* < 0.01, *** *p* < 0.001. Data are pooled from three independent experiments with n = 5 mice per group.

Given the anti-inflammatory and immunosuppressive functions of metabolite SCFAs, Luminex cytokine microarray was conducted to detect the cytokines profile changes in intestinal mucosa following PTL administration. The differentially expressed cytokines (DECs) mediated through PTL administration were displayed by the volcano plot (Figure 6A), and the top 20 DECs were shown by the heatmap (Figure 6B). Compared with the DSS group, the pro-inflammatory cytokines manifested significantly decreasing trend in the PTL+DSS group, especially IL-1β, TNF-α, IL-12, IL-6, IFN-γ, IL-17A, RANTES, and IL-1α. Instead, the anti-inflammatory cytokines, including IL-4 and IL-10, were significantly increased in the PTL+DSS group (Figure 6A-B). The protein levels of 20 DECs in colon tissue were measured through ELISA (data not shown). Among them, IL-1β, TNF-α, IL-6, IL-10 and IL-17A manifested significant differences between the two groups (Figure 6C), which were consistent with the cytokine microarray results. Notably, the immunosuppressive cytokine IL-10 displayed an approximately 5.52-fold increase in colon tissue in mice that received PTL treatment compared with the DSS group.

The cytokines profile in colon tissue were also conducted in gut microbiota-depletion groups. Strikingly, the levels of proinflammatory cytokines, including IL-1β, TNF-α, IL-6, and IL-17A, and the anti-inflammatory cytokine IL-10 exhibited similar levels, and there was no significant difference between the ABX(DSS) and ABX(PTL+DSS) groups (*p* > 0.05) (Figure 6D). Metabolomics results indicated that FMT increased metabolite SCFAs, so we supposed that FMT can also affect the cytokines profile. As expected, colon tissue from FM(PTL) → GD WT showed less protein levels of IL-1β, TNF-α, IL-6, and IL-17A but higher IL-10 compared with colons from FM(DSS) → GD WT (Figure 6E). In conclusion, PTL administration markedly altered the cytokines profile through downregulating the level of pro-inflammatory cytokines as well as upregulating the immunosuppressive cytokines in DSS-induced colitis.

### PTL shaped intestinal immune responses through regulating Treg/Th17 balance

**Figure 7:**
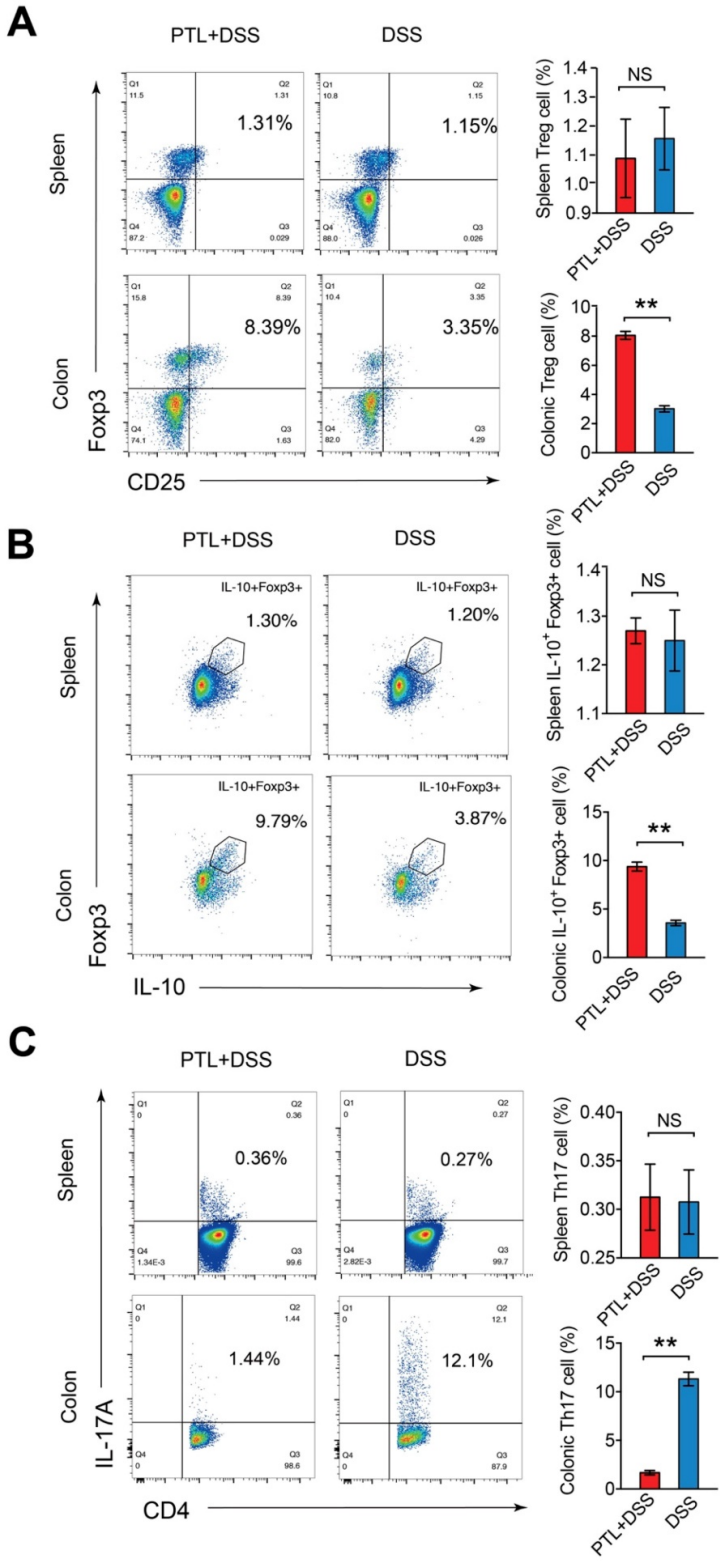
PTL treatment altered the frequency of Treg cells and Th17 cells in LP. (A) CD4^+^CD25^+^Foxp3^+^ (Treg) cells in the colonic LP and spleen from PTL+DSS group and DSS group were analyzed by flow cytometry and bar charts of the percentage of Treg cells. (B) Representative plot of IL-10^+^Foxp3^+^ cells in the colonic LP and spleen from PTL+DSS and DSS groups and bar charts of the percentage of IL-10^+^Foxp3^+^ cells. (C) Representative plot of CD4^+^IL-17A^+^ (Th17) cells in the colonic LP and spleen from PTL+DSS and DSS groups and bar charts of the percentage of Th17 cells. Plot numbers represent the percentage of CD4^+^ T-cells in the respective quadrants. (A-C) n = 6 mice per group. Data are shown as mean values ± SD are presented, *p* values were calculated using Unpaired T-test, * *p* < 0.05, ** *p* < 0.01, *** *p* < 0.001. Data are pooled from three independent experiments with n = 6 mice per group.

Given the markedly altered cytokines profile after PTL administration, immunosuppressive cytokine of IL-10 and pro-inflammatory cytokine of IL-17A manifested the most significant difference. It is well believed that Treg/Th17 balance in intestinal mucosa plays a fundamental role in stabilizing gut microecosystem homeostasis. To investigate the impact of PTL administration on Treg/Th17 balance, T cell responses in the colonic lamina propria (LP) were phenotyped by flow cytometry. The relative abundance of each cell type was reported as a percent of the overall CD4^+^ T cells, generating a community data matrix for ecological analyses. As shown in Supplementary Figure S4, Th1(72.64% vs 76.89%; *p* > 0.05) and Th2 (6.65% vs 7.10%; *p* > 0.05) cell responses displayed no significant difference following PTL administration. However, analysis of the CD4^+^ T cell compartment for CD25 and Foxp3 expression revealed the percentage of Treg cells in the PTL+DSS group was 2.67-fold higher than that in the DSS group (8.06% vs 3.01%; *p* < 0.05) (Figure 7A). Consistently, the PTL+DSS group had higher absolute number of Treg cells compared to the DSS group (*p* < 0.05) (Supplementary Figure S5A).

As IL-10 is the key cytokine in Treg-mediated inflammatory suppression and the elevation of IL-10 production in colon tissue was observed in previous cytokine measurement results (Figure 6C), the frequency and absolute number of IL-10^+^ Foxp3^+^ cells were also evaluated. The ratio of IL-10^+^ Foxp3^+^ cells isolated from colonic LP in the PTL+DSS group was 2.63-fold higher than that in the DSS group (9.38% vs 3.57%; *p* < 0.05) (Figure 7B), with a corresponding trend in the absolute number of IL-10^+^ Foxp3^+^ cells (*p* < 0.05) (Supplementary Figure S5B). However, analysis of the CD4^+^ T cell compartment for IL-17A expression revealed that the frequency of Th17 cells in the PTL+DSS group was significantly lower than that in the DSS group (1.67% vs 11.32%; *p* < 0.05) (Figure 7C), which was consistent with the decreased IL-17A concentration in colon tissue after PTL administration (Figure 6C). The absolute counts of Th17 cells in the PTL+DSS group were significantly lower than that in the DSS group (*p* < 0.05) (Supplementary Figure S5C). To investigate whether PTL treatment exerted an influence on systemic immune responses, the proportion Treg cells (1.09% vs 1.16%; *p* > 0.05) (Figure 7A), IL-10^+^ Foxp3^+^ cells (1.27% vs 1.25%; *p* > 0.05) (Figure 7B) and Th17 cells (0.31% vs 0.31%; *p* > 0.05) (Figure 7C) in mice spleen were also analyzed. The absolute numbers of these T lymphocyte subsets in mice spleen also displayed no significant difference between groups (*p* > 0.05) (Supplementary Figure S5A-5C). In summary, PTL administration selectively upregulated the frequency of colonic Treg cells as well as downregulated the ratio of colonic Th17 cells against DSS-induced colitis, improving the Treg/Th17 balance to maintain intestinal homeostasis.

### PTL regulated Treg/Th17 balance in a gut microbiota-dependent manner

**Figure 8:**
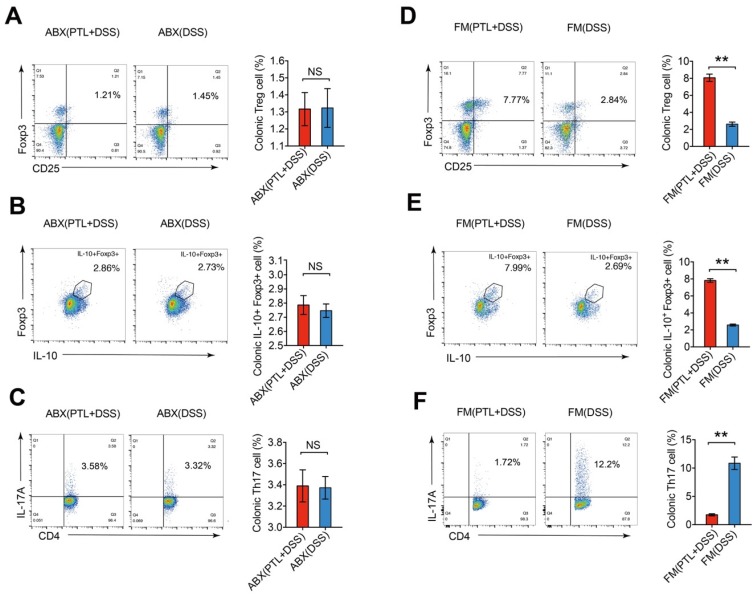
The frequency of Treg cells and Th17 cells in mice LP after gut microbiota depletion and FMT. (A) Treg cells in the colonic LP from ABX(PTL+DSS) and ABX(DSS) group were analyzed by flow cytometry and bar charts of the percentage of Treg cells were displayed. (B) Representative plot and graph analysis of IL-10^+^Foxp3^+^ cells in the colonic LP from ABX(PTL+DSS) and ABX(DSS) groups. (C) Representative plot and graph analysis of Th17 cells in the colonic LP from ABX(PTL+DSS) and ABX(DSS) groups. (D) Treg cells in the colonic LP from FM(PTL+DSS) and FM(DSS) group were analyzed by flow cytometry and bar charts of the percentage of Treg cells were displayed. (E) Representative plot and graph analysis of IL-10^+^Foxp3^+^ cells in the colonic LP from FM(PTL+DSS) and FM(DSS) groups. (F) Representative plot and graph analysis of Th17 cells in the colonic LP from FM(PTL+DSS) and FM(DSS) group. Plot numbers represent the percentage of CD4^+^ T cells in the respective quadrants. (A-F) n = 6 mice per group. Data are shown as mean values ± SD are presented, *p* values were calculated using Unpaired T-test, * *p* < 0.05, ** *p* < 0.01, *** *p* < 0.001. Data are pooled from three independent experiments with n = 6 mice per group.

To further investigate whether the improved Treg/Th17 balance after PTL administration was gut microbiota-dependent, colonic LP cells isolated from gut microbiota depletion groups were phenotyped by flow cytometry. The percentage of Treg cells (1.32% vs 1.32%; *p* > 0.05) (Figure 8A), IL-10^+^ Foxp3^+^ cells (2.79% vs 2.75%; *p* > 0.05) (Figure 8B) and Th17 cells (3.39% vs 3.37%; *p* > 0.05) (Figure 8C) displayed no significant difference between the ABX(PTL+DSS) and ABX(DSS) groups. Consistently, the absolute numbers of these T lymphocyte subsets manifested corresponding trends in gut microbiota depletion groups (*p* > 0.05) (Supplementary Figure S6A).

Meanwhile, the frequencies and numbers of Treg cells and Th17 cells in colonic LP were also phenotyped in the FMT groups. The percentage of Treg cells in the FM(PTL+DSS) group was 3.08-fold greater than that in the FM(DSS) group (8.07% vs 2.62%; *p* < 0.05) (Figure 8D). The FM(PTL+DSS) group had higher absolute number of Treg cells compared to the FM(DSS) group (*p* < 0.05) (Supplementary Figure S6B). The ratio of IL-10^+^ Foxp3^+^ cells in the FM(PTL+DSS) group was 3.04-fold higher than that in the FM(DSS) group (7.82% vs 2.57%; *p* < 0.05) (Figure 8E), which displayed a corresponding trend to the elevation of the cytokine IL-10 levels in the FM(PTL+DSS) group (Figure 6E). The absolute counts of IL-10^+^ Foxp3^+^ cells in the FM(PTL+DSS) group were significantly higher than that in the FM(DSS) group (*p* < 0.05) (Supplementary Figure S6B). In contrast, the ratio of Th17 cells in the FM(PTL+DSS) group was significantly lower than that in the FM(DSS) group (1.75% vs 10.85%; *p* < 0.05) (Figure 8F), with a consistent tendency of the absolute Th17 cells counts between groups (*p* < 0.05) (Supplementary Figure S6B). These results indicated that the altered gut microbiota following PTL administration were responsible for the improved Treg/Th17 balance in intestinal mucosa. In summary, PTL regulated Treg/Th17 balance in a gut microbiota-dependent manner.

## Discussion

In this study, the impact of PTL on colon inflammation was investigated *in vivo*. Our results indicated the significant protective effect of PTL on DSS-induced colitis, as evidenced by a reduction in body weight loss, mortality, DAI score, shortening of colon length as well as decreased histology score and colonoscopy score. We also demonstrated that PTL alleviated colon inflammation in a gut microbiota-dependent manner through gut microbiota depletion. Gut microbiota exhibited more abundant microbial diversity and flora composition after PTL administration, which is beneficial for SCFAs production, thus improving the Treg/Th17 balance in the intestinal mucosa. FMT was also conducted to confirm this gut microbiota-dependent mechanism. Our results support that PTL is a promising natural plant extract for the prevention and treatment of IBD through gut microbiota modulation.

Among various chemically induced colitis models, the DSS-induced colitis model is widely used because of its simplicity and many similarities with human ulcerative colitis 34, 43. Consistent with human IBD pathogenesis, gut microbiota dysbiosis associated with immune response is deeply involved in DSS-induced colitis. As such, DSS-induced colitis is an ideal model for studying drug therapy strategies targeting gut microbiota disorders. Acute, chronic, and relapsing models of intestinal inflammation can be achieved by modifying the concentration of DSS and the frequency of administration. Therefore, we used 3.0% DSS for acute inflammation induction, but when the antibiotic cocktails were administered for gut microbiota depletion, we reduced the concentration to 1.5% to prevent pre-antibiotic-treated mice from experiencing enhanced sensitivity to DSS 37.

Since PTL has been reported to have significant anti-inflammatory effects *in vitro* and its mechanism includes specific inhibition of the NF-kB pathway 27, the anti-inflammatory and anti-bacterial effects *in vivo*, especially in IBD, have rarely been described. Therefore, we first studied the therapeutic effect of PTL on IBD *in vivo.* As expected, significantly relieved colon inflammation was achieved with PTL administration. Distinguished from common inflammatory diseases, gut microbiota dysbiosis plays an indispensable pathogenic role in IBD. Here, it was necessary to study whether gut microbiota participate in the protective effect of PTL on colitis. Strikingly, the protective effect of PTL on colon inflammation disappeared following gut microbiota depletion, and the severity of colon inflammation between ABX(PTL+DSS) and ABX(DSS) groups displayed no significant difference. Subsequently, we performed FMT to confirm the above observations. The wild-type mice reconstituted with the microbiota of PTL-treated mice (FM(PTL+DSS) → GD WT) had alleviated colitis symptoms and intestinal injuries compared with DSS-treated mice (FM(DSS) → GD WT). The protective effect of PTL on colon inflammation appeared after FMT, and colitis severity between the FM(PTL+DSS) and FM(DSS) groups manifested a remarkable difference. These results indicated that the altered gut microbiota after PTL administration was responsible for alleviated colon inflammation. It is worth exploring whether PTL also exerts anti-inflammatory effects in a gut microbiota-independent manner *in vitro,* as other evidence has shown, but in IBD, it may be more effective in modulating the gut microbiota to mask its anti-inflammatory effects through other signaling pathways. More in-depth and precise experiments need to be designed to investigate the specific mechanism involved.

The patients with IBD showed reduced biodiversity in gut microbial composition called dysbiosis, characterized by the loss of beneficial commensal microflora and the expansion of pathogenic bacteria 8. Transfer of more than 30 different Clostridium human strains into germ-free mice caused a three-fold expansion of Treg cells, while the transfer of a single strain from the same Clostridium collection resulted in a modest Treg response, suggesting that a larger microbial diversity could maintain host immune response homeostasis and that biodiversity reduction may lead to the inflammatory processes of IBD 44. We used 16S rRNA sequencing to investigate the potential alterations in microbiological diversity and composition after PTL treatment. Alpha diversity measurement results, based on OTUs, Chao, Shannon and Simpson indexes, showed that PTL-treated mice exhibited a microbiota with significantly higher diversity relative to that of the DSS-treated group. Regarding beta diversity, PTL+DSS group mice harbored an apparent clustering separation from DSS group mice through PCoA, indicating that PTL treatment markedly transformed the biological community structures. To identify the underlying biomarkers and dominant bacteria mediated through PTL treatment, LEfSe analysis was conducted between PTL+DSS and DSS groups. Importantly, 3 signature bacterial taxa, including *Alloprevotella, Rikenella,* and* Fournierella,* displayed a relative enrichment in the PTL+DSS group, which were all described to be beneficial for gut microbiota metabolite SCFAs production 38-40. Additionally, the genus *Alloprevotella,* with the most predominance and the highest LDA score in the PTL+DSS group, was reported to be associated with decreased lifetime cardiovascular disease risk 41. Therefore, our observations demonstrated that PTL administration could modulate gut microbiota dysbiosis caused by DSS-induced colitis through increasing intestinal flora biological diversity and promoting the relative abundance of beneficial bacteria.

Recent studies have shown that intestinal microbial community composition and bacterial metabolites can significantly improve the outcome of IBD through regulating inflammatory responses 21, 45. In particular, microbiota-derived SCFAs play an essential role in stabilizing intestinal immune homeostasis through their anti-inflammatory and immune-suppressive functions 19. Given that the predominant bacteria in the PTL+DSS group associated with SCFAs metabolism and decreased SCFAs concentrations were observed in patients with IBD as well as in DSS-induced colitis in mouse models 20, 42 , SCFAs concentrations in cecal contents and fecal samples between the PTL+DSS and DSS groups were evaluated. Targeted metabolomics results showed that PTL administration significantly improved the production of microbiota-derived SCFAs. Additionally, FMT also alleviated colon inflammation and increased microbiota-derived SCFAs.

Previous studies illuminated that SCFAs could regulate the size and function of the colonic Treg pool and protect against DSS-induced colitis in mouse models 18. Expressing the specific transcription factor Foxp3, Treg cells have been identified as dedicated suppressors of diverse immune responses and inflammation through inhibition of other immune cells, such as Th17 cells, and secretion of the anti-inflammatory cytokine IL-10. Additionally, PTL administration markedly altered the cytokines profile through downregulating the level of pro-inflammatory cytokines as well as upregulating the immunosuppressive cytokines in DSS-induced colitis. In particular, immunosuppressive cytokine of IL-10 and pro-inflammatory cytokine of IL-17A manifested the most significant difference. The dynamic Treg/Th17 balance plays an integral role in stabilizing gut microecosystem homeostasis, which can be regulated through microbiota-derived SCFAs signals 10, 11. In our study, T cell responses in the colonic lamina propria were investigated by flow cytometry. Th1 and Th2 cell responses displayed no significant difference between two groups. As expected, increased metabolite SCFAs production mediated through PTL administration dramatically improved the Treg/Th17 balance in intestinal mucosa in PTL+DSS group, manifested by the increased frequency of Treg cells and IL-10^+^Foxp3^+^ T cells as well as decreased Th17 cells compared with the DSS group. To further testify the promoted Treg/Th17 balance in intestinal mucosa, the absolute numbers of these T lymphocyte subsets were calculated. Consistently, the absolute numbers of Treg cells and Th17 cells displayed corresponding trend between the PTL+DSS and DSS groups. After gut microbiota depletion, the ratio and absolute counts of Treg cells and Th17 cells displayed no significant difference between the ABX(PTL+DSS) and ABX(DSS) groups. These data indicated that PTL regulated Treg/Th17 balance in a gut microbiota-dependent manner. Additionally, when GD WT mice were transplanted with fecal microbiota of PTL-treated mice, the levels of metabolite SCFAs increased consistently. The improved Treg/Th17 balance in intestinal mucosa can be manifested through the increased frequency and numbers of Treg cells, while the decreased ratio and counts of Th17 cells in the FM(PTL+DSS) group. Our findings indicated that the altered fecal microbiota following PTL administration resulted in alleviated colon inflammation mediated through increased metabolite SCFAs productions, which improved the Treg/Th17 balance during IBD progression. Some researchers believe that intestinal inflammation in DSS-induced acute colitis is due to epithelial barrier injury and innate immune response activation. Therefore, we also analyzed T cell responses in chronic colitis models (Supplementary Figure S7). Strikingly, T cell responses in chronic colitis models manifested similar tendency in acute colitis models, which indicated that the improved Treg/Th17 balance in intestinal mucosal was mediated through PTL administration in both acute and chronic colitis models.

In conclusion, our data demonstrated that PTL ameliorated colon inflammation in a gut microbiota-dependent manner. The underlying protective mechanism was associated with the improved Treg/Th17 balance in intestinal mucosa mediated through increased microbiota-derived SCFAs productions. Collectively, our results demonstrated the role of PTL as a potential gut microbiota modulator to prevent and treat colon inflammation. However, although PTL is a promising drug for the prevention and treatment of IBD, its potential mechanism through which it affects the gut microbiota needs further study.

## Supplementary Material

Supplementary figures and table.

Supplementary video 1.

Supplementary video 2.

## Abbreviations

IBD: inflammatory bowel disease
PTL: parthenolide
DSS: dextran sodium sulfate
DAI: disease activity index
SCFAs: short-chain fatty acids
LP: lamina propria
IL-6: interleukin-6
IL-1β: interleukin-1β
IL-17A: interleukin-17A
IL-10: interleukin-10
Treg: regulatory T
Th17: T helper type 17
FMT: fecal microbiota transplantation
FM: fecal microbiota
CD: Crohn's disease
UC: ulcerative colitis
CAC: colitis-correlated colorectal cancer
GD: gut microbiota-depleted
PCoA: principal coordinate analysis
ELISA: Enzyme-linked immunosorbent assay
