# Supplementary material for: Parthenolide ameliorates colon inflammation through regulating Treg/Th17 balance in a gut microbiota-dependent manner
Source: Theranostics. 2020 Apr 6;10(12):5225–41. doi: 10.7150/thno.43716 (PMC7196297; doi:10.7150/thno.43716)
Supplement: Supplementary file 1 — Supplementary figures and table. [file thnov10p5225s1.pdf]

Supplementary Table S1

Scoring system for Disease Activity Index (DAI)

Score	Weight loss	Stool consistency	Blood stool
0	no loss	normal	no blood
1	1-5%	loose stool	
2	5-10%	watery diarrhea	presence of blood
3	10-20%	slimy diarrhea, little blood	
4	> 20%	severe watery diarrhea with blood	gross bleeding

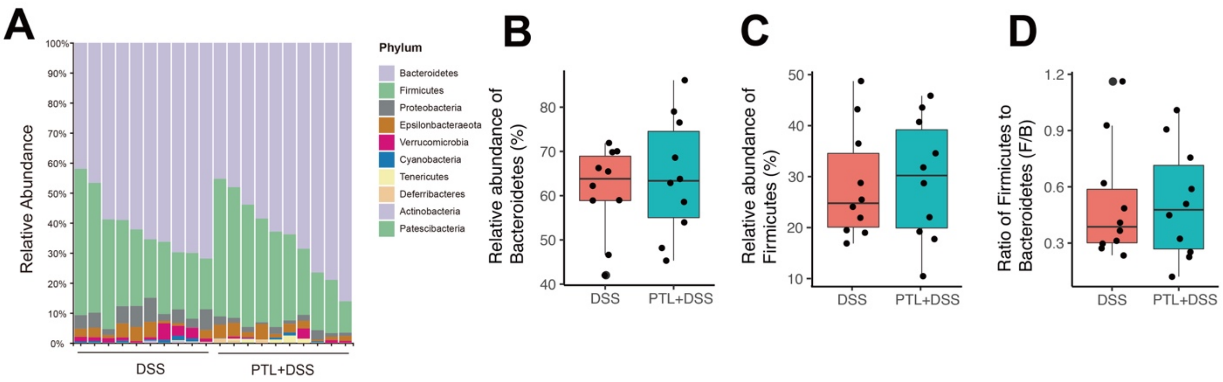

Supplementary Figure S1.

(A) Bar plots of the phylum taxonomic levels in DSS and PTL+DSS group. Relative abundance is plotted for each sample. (B) The relative abundances of Bacteroidetes. (C) The relative abundances of Firmicutes. (D) The ratio of Firmicutes to Bacteroidetes (F/B).

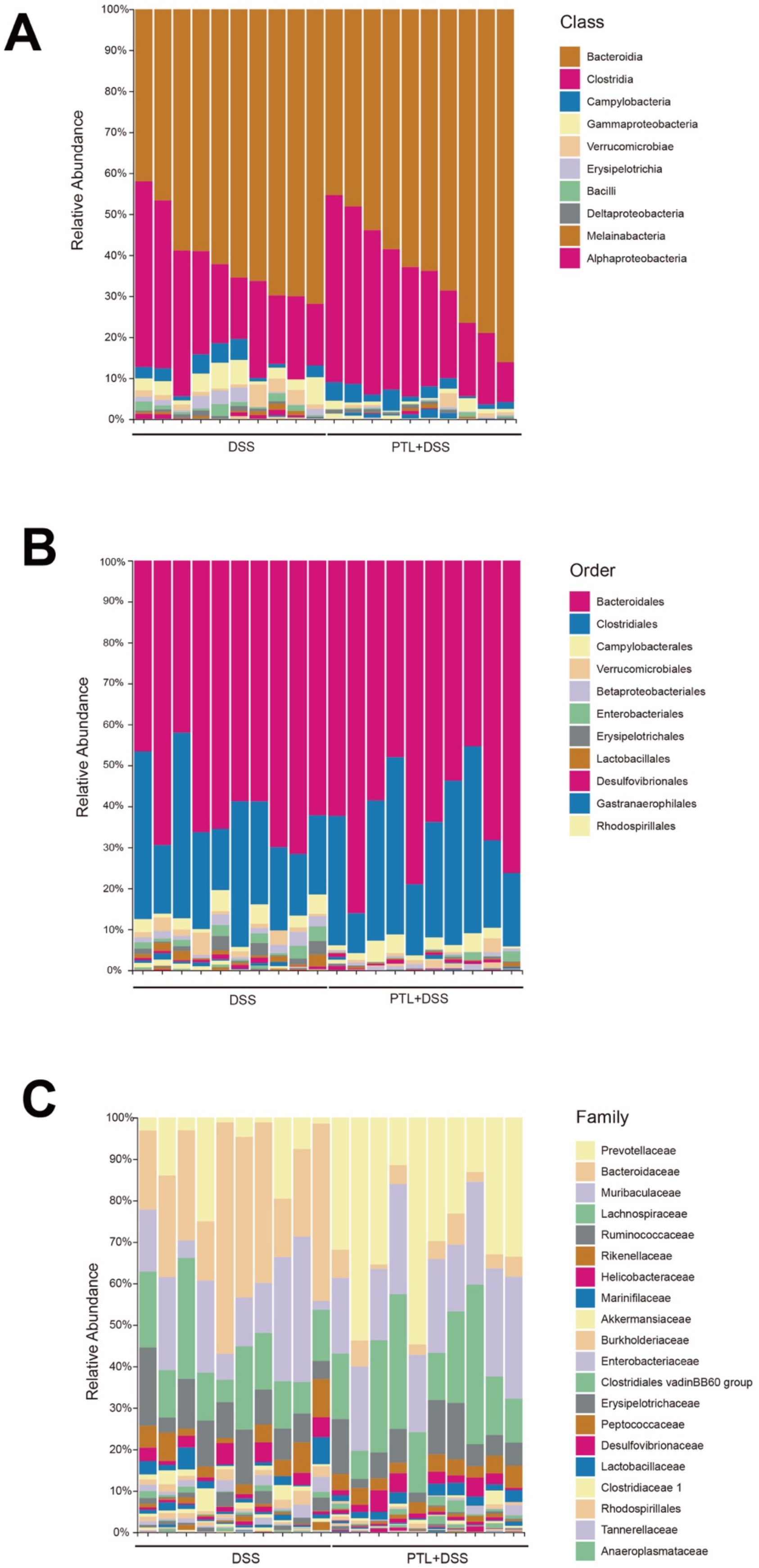
A
100%
90%
80%
70%
60%
50%
40%
30%
20%
10%
0%
Relative Abundance
DSS
PTL+DSS
Class
Bacteroidia
Clostridia
Campylobacteria
Gammaproteobacteria
Verrucomicrobiae
Erysipelotrichia
Bacilli
Deltaproteobacteria
Melainabacteria
Alphaproteobacteria
B
100%
90%
80%
70%
60%
50%
40%
30%
20%
10%
0%
Relative Abundance
DSS
PTL+DSS
Order
Bacteroidales
Clostridiales
Campylobacterales
Verrucomicrobiales
Betaproteobacteriales
Enterobacteriales
Erysipelotrichales
Lactobacillales
Desulfovibrionales
Gastranaerophilales
Rhodospirillales
C
100%
90%
80%
70%
60%
50%
40%
30%
20%
10%
0%
Relative Abundance
DSS
PTL+DSS
Family
Prevotellaceae
Bacteroidaceae
Muribaculaceae
Lachnospiraceae
Ruminococcaceae
Rikenellaceae
Helicobacteraceae
Marinifilaceae
Akkermansiaceae
Burkholderiaceae
Enterobacteriaceae
Clostridiales vadinBB60 group
Erysipelotrichaceae
Peptococcaceae
Desulfovibrionaceae
Lactobacillaceae
Clostridiaceae 1
Rhodospirillales
Tannerellaceae
Anaeroplasmataceae

Supplementary Figure S2.

(A) Bar plots of the class taxonomic levels in DSS and PTL+DSS group. Relative abundance is plotted for each sample. (B) Bar plots of the order taxonomic levels in DSS and PTL+DSS group. Relative abundance is plotted for each sample. (C) Bar plots of the family taxonomic levels in DSS and PTL+DSS group. Relative abundance is plotted for each sample.

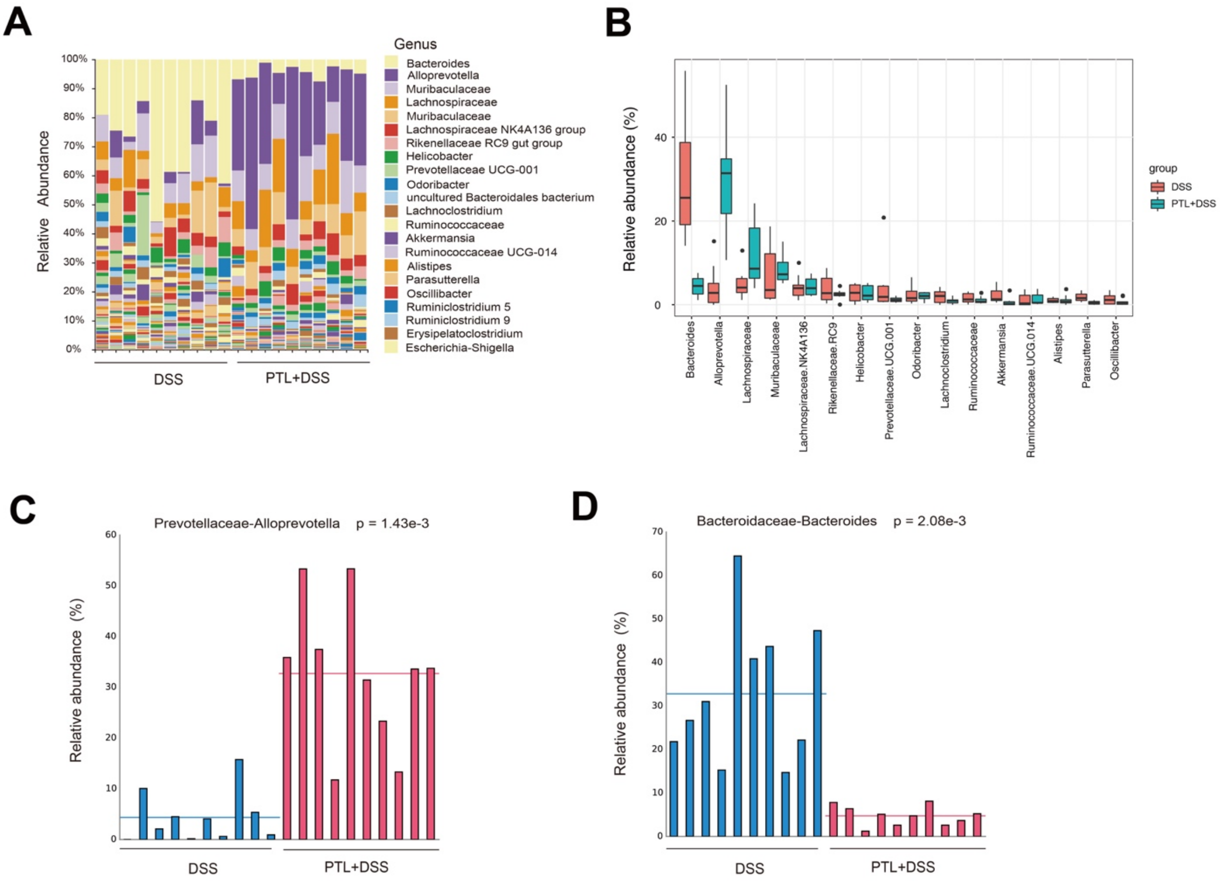

Supplementary Figure S3.

(A) Bar plots of the genus taxonomic levels in DSS and PTL+DSS group. Relative abundance is plotted for each sample. (B) Bar plots comparing taxonomic composition (genus level), and bacterial genera that were present at a relative abundance of > 1% were analyzed. (C) Relative abundance of genus *Alloprevotella* in each sample were displayed by bar plots. (D) Relative abundance of genus *Bacteroides* in each sample were displayed by bar plots.

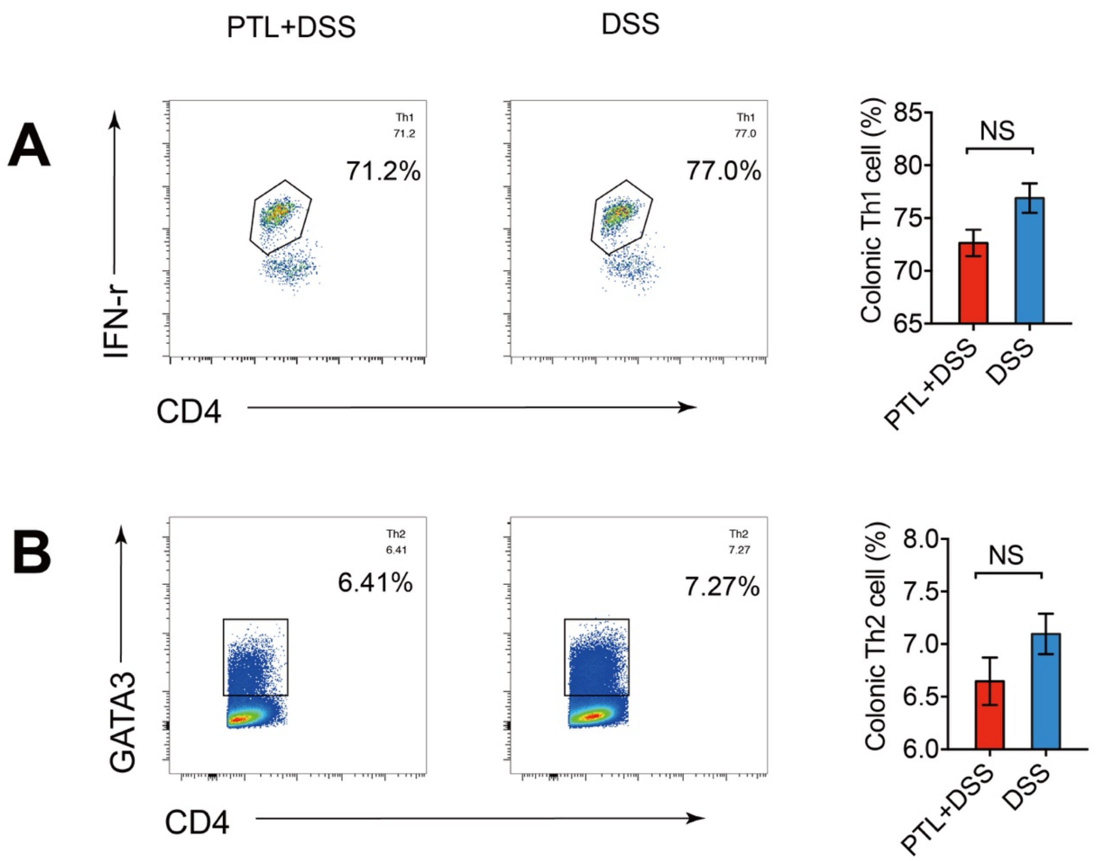

Supplementary Figure S4.

(A) Th1 cells in the colonic LP from PTL+DSS and DSS groups were analyzed by flow cytometry and bar charts of the percentage of Th1 cells were displayed. (B) Representative plot and graph analysis of Th2 cells in the colonic LP from PTL+DSS and DSS groups. n = 6 mice per group. Data are shown as mean values ± SD are presented, *p* values were calculated using Unpaired T-test, * $p < 0.05$, ** $p < 0.01$, *** $p < 0.001$.

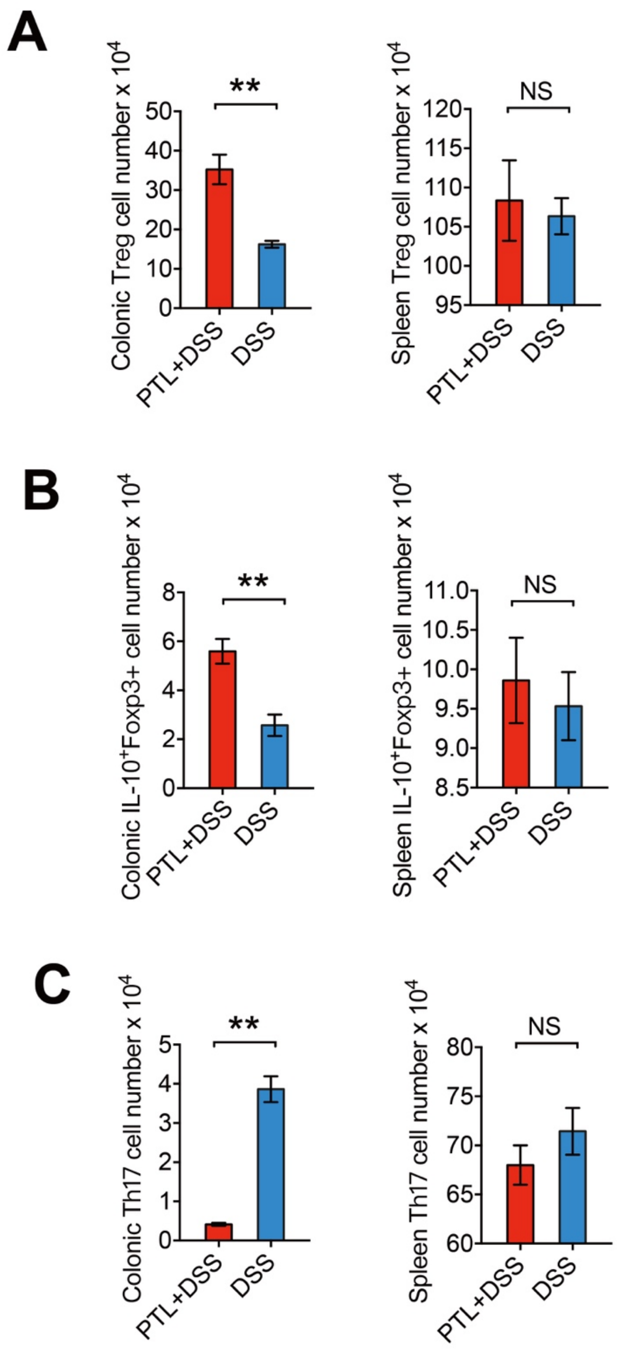

Supplementary Figure S5.

(A) Treg cells numbers in the colonic LP and spleen from PTL+DSS and DSS groups were calculated. (B) IL-10^{+}Foxp3^{+} cells numbers in the colonic LP and spleen from PTL+DSS and DSS groups were analyzed. (C) Th17 cells numbers in the colonic LP and spleen from PTL+DSS and DSS groups were calculated. n = 6 mice per group. Data are shown as mean values ± SD are presented, p values were calculated using Unpaired T-test, * $p < 0.05$, ** $p < 0.01$, *** $p < 0.001$.

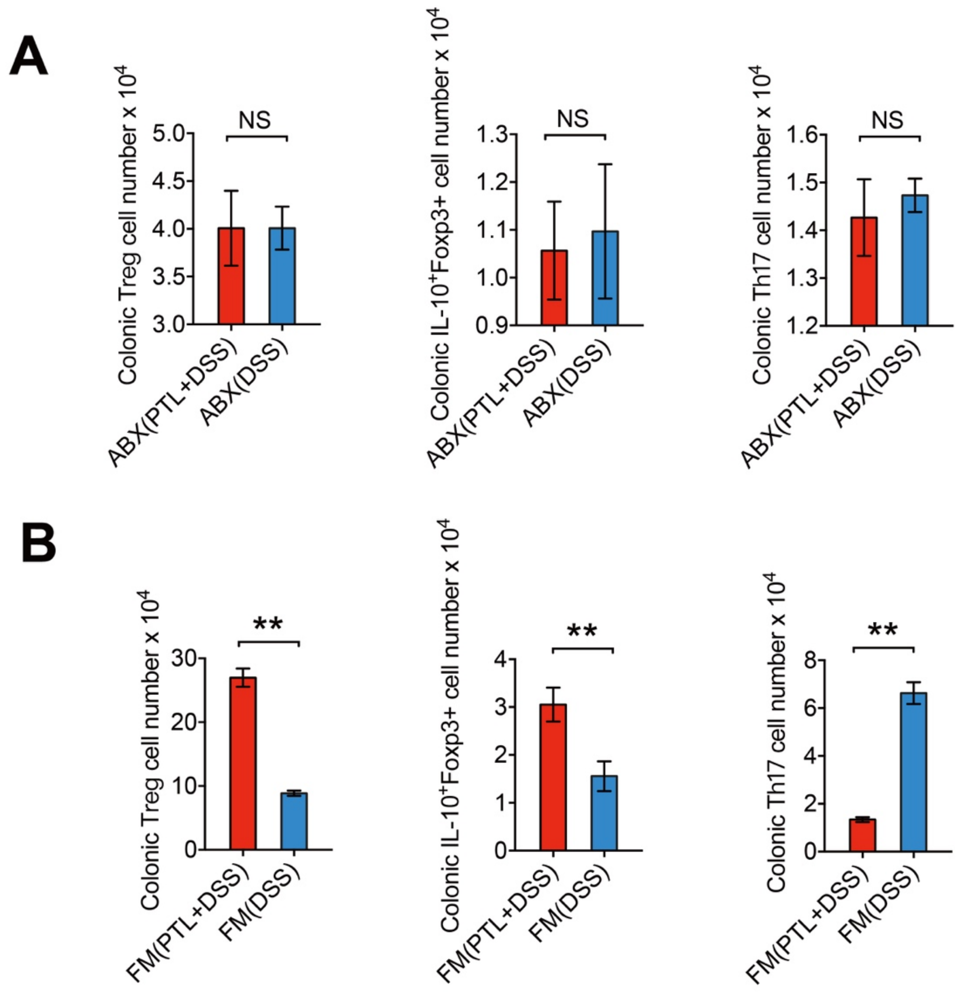

Supplementary Figure S6.

(A) Treg cells, IL-10^{+}Foxp3^{+} cells, Th17 cells numbers in the colonic LP from ABX(PTL+DSS) and ABX(DSS) group were analyzed. (B) Treg cells, IL-10^{+}Foxp3^{+} cells, Th17 cells numbers in the colonic LP from FM(PTL+DSS) and FM(DSS) groups. n = 6 mice per group. Data are shown as mean values ± SD are presented, p values were calculated using Unpaired T-test, * $p < 0.05$, ** $p < 0.01$, *** $p < 0.001$.

Chronic colitis model

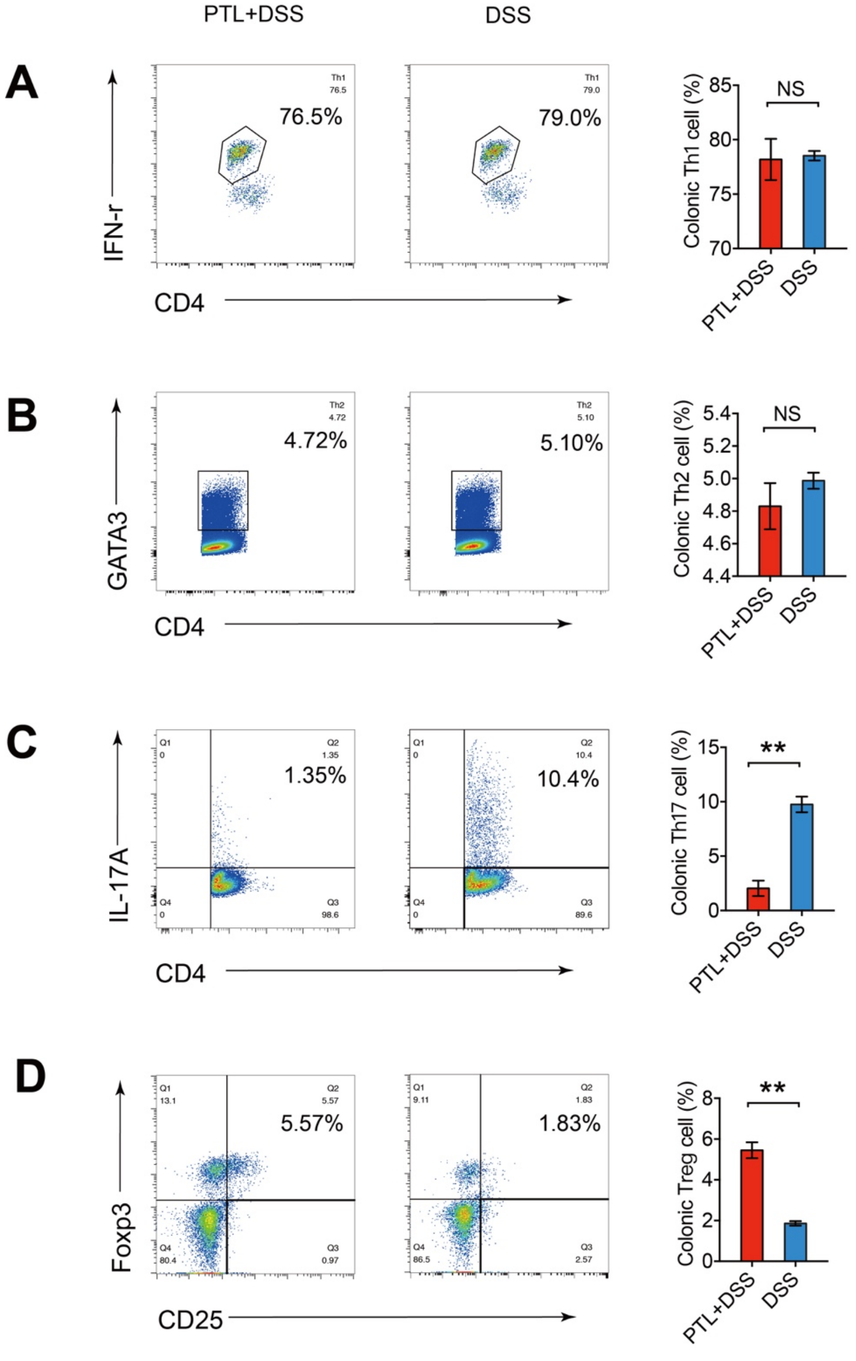

Supplementary Figure S7.

(A) Th1 cells in the colonic LP from PTL+DSS and DSS groups were analyzed by flow cytometry and bar charts of the percentage of Th1 cells were displayed. (B) Representative plot and graph analysis of Th2 cells in the colonic LP from PTL+DSS and DSS groups. (C) Th17 cells in the colonic LP from PTL+DSS and DSS groups were analyzed by flow cytometry and bar charts of the percentage of Th17 cells were displayed. (D) Representative plot and graph analysis of Treg cells in the colonic LP from PTL+DSS and DSS groups. n = 6 mice per group. Data are shown as mean values ± SD are presented, p values were calculated using Unpaired T-test, * $p < 0.05$, ** $p < 0.01$, *** $p < 0.001$.
